# Barriers and facilitators for treatment-seeking in adults with a depressive or anxiety disorder in a Western-European health care setting: a qualitative study

**DOI:** 10.1186/s12888-022-03806-5

**Published:** 2022-03-05

**Authors:** Ruth C. Waumans, Anna D. T. Muntingh, Stasja Draisma, Klaas M. Huijbregts, Anton J. L. M. van Balkom, Neeltje M. Batelaan

**Affiliations:** 1 Amsterdam UMC, Vrije Universiteit (VU) Psychiatry, Amsterdam Public Health Research Institute, Amsterdam, The Netherlands; 2 GGZ inGeest Specialised Mental Health Care, Amsterdam, The Netherlands; 3 GGNet, Mental Health – RGC Winterswijk, Winterswijk, The Netherlands

**Keywords:** Adults, Depression, Anxiety, Qualitative research, Thematic analysis, Framework analysis, Treatment-seeking

## Abstract

**Background:**

Previous research on barriers and facilitators regarding treatment-seeking of adults with depressive and anxiety disorders has been primarily conducted in the Anglosphere. This study aims to gain insight into treatment-seeking behaviour of adults with depressive and anxiety disorders in a European healthcare system.

**Methods:**

In-depth semi-structured interviews were conducted with 24 participants, aged ≥18 years and diagnosed with an anxiety disorder and/or depressive disorder according to DSM-IV. Participants were purposively sampled from an outpatient department for mental health care in the Netherlands. The seven steps of framework analysis were used to identify relevant themes emerging from the interviews.

**Results:**

Data analysis suggested an interplay between individual aspects, personal social system, healthcare system and sociocultural context influences. Amongst the most relevant themes were mental health illiteracy, stigma, a negative attitude toward professional help, the influence of significant others and general practitioner, and waiting time. Financial barriers were not of relevance.

**Conclusions:**

Even in a country with a well-developed mental health care system and in absence of financial barriers, there are many barriers to treatment-seeking in adult patients with depressive and anxiety disorders. National campaigns to increase awareness and decrease stigma in the general population, and to empower the social environment might reduce the treatment gap.

**Supplementary Information:**

The online version contains supplementary material available at 10.1186/s12888-022-03806-5.

## Background

People with an anxiety or depressive disorder often experience a long delay between the expression of their symptoms and receiving treatment. According to the World Health Organization (WHO), the delay for eventually making treatment contact ranged from 1 to 14 years in patients with mood disorders, and from 3 up to 30 years in those with anxiety disorders worldwide [1]. Even in countries with high quality mental health care and/or a strong primary health care system [2, 3], 48-89% of the adults did not seek professional help in the first year following the onset of an anxiety or depressive disorder ( [1, 4]). Treatment-seeking was also limited in subsequent years, as 61% did not seek treatment in the three subsequent years either [4]. Treatment gap and delays result in a longer duration of untreated illness, which is associated with multiple adverse consequences, including a higher risk to develop comorbidity [5, 6], a limited response to medication [6, 7], and a higher risk of unfavourable outcome [8, 9].

To reduce treatment gap and delay and its undesired consequences, insight in barriers and facilitating factors regarding treatment-seeking is crucial. Among the risk factors associated with impeded treatment-seeking are relatively stable factors such as socio-demographics (e.g., young or old age, male gender and certain ethnic minorities) [10–12]. This information might be helpful in identifying groups that do not easily reach mental health care. Other factors, including attitudes and organisational aspects [12, 13], may be modifiable. Identifying these factors might generate ideas on how to facilitate treatment-seeking on population level and hence, to overcome the negative consequences of long episodes of untreated illness.

Previous research concluded that lack of knowledge about mental disease and about treatment itself substantially hinders treatment-seeking [13–17]. In line with this, Nuijen and colleagues [4] report that recognition of one’s psychological problems facilitated treatment-seeking. Sareen [18] found attitudinal barriers to be of primary importance. Frequently reported attitudinal barriers are the wish to solve the problem oneself [14, 18–21], the idea that symptoms would remit spontaneously [14, 18] and the belief that mental health care would not help [13, 18, 19]. A recent systematic review [22] of both quantitative and qualitative studies on stigma among participants with various mental disorders identified stigma as a barrier to access mental healthcare in general. Having a more severe disorder, having multiple depressive and/or anxiety disorders, and worse social functioning were all positively associated with treatment-seeking [4]. Finally, previous studies [12, 16, 20, 21] reported that financial aspects of mental health treatment can interfere with access to mental health care.

However, the majority of these studies is from the Anglosphere, (i.e. the United States (US), United Kingdom (UK) and Canada) [12, 13], limiting ecological validity for European countries. Among other European countries, the Netherlands has a high-access healthcare system including a strong primary health care system [2] free of charge, with the general practitioner as an important gatekeeper and a mental health nurse readily available in most primary care centres. In addition, mental health care is almost fully covered for all inhabitants. This makes it interesting to investigate whether previously identified factors are also relevant in a high-access setting where costs for health care are barely an issue [23], and whether unique factors play a role. Qualitative research offers the opportunity to gain rich in-depth information and hence promotes understanding of complex topics, leading to deepening insight on factors influencing treatment-seeking. Therefore, the aim of this study was to examine both barriers and facilitating factors of treatment-seeking in patients with anxiety or depressive disorders in a Western-European healthcare setting, using qualitative methods.

## Methods

### Design

In this qualitative study we used semi-structured interviews to gain insight into facilitators and barriers of help-seeking in participants with anxiety and/or depressive disorders who sought treatment in mental health care. With a multidisciplinary research team, a stepwise framework analysis method was applied to order the data from the semi-structured interviews. Framework analysis [24, 25] offers steps and procedures to attain structured output of summarized qualitative data, suitable for team research. This manuscript was written according to the Consolidated criteria for Reporting Qualitative research (COREQ) checklist [26]. The study protocol was approved by the Medical Ethics Review Committee of VU University Medical Centre.

### Sampling and recruitment

Participants were purposively sampled from an outpatient department for mental health care in the Netherlands, with several locations. Therapists at the outpatient department included psychologists, psychiatrists and psychiatrists in training, offering evidence-based treatments for anxiety and/or depressive disorders, e.g., cognitive behaviour therapy and medication. The recruitment of new participants aimed at including a diverse study population with respect to age, gender, ethnicity and specific diagnosis. Newly referred patients who during their first appointments were diagnosed with a DSM-IV anxiety and/or depressive disorder were invited in a face-to-face session by their therapist to participate in this study. The therapist explained the study aim and procedure and referred to the researchers for additional information. Of those who were willing to participate, contact details were shared with the researchers*.* After the information was provided, the participant received an invitation for the face-to-face research interview. Participation in this study was voluntary. Participation nor refusal influenced the diagnostic and treatment procedures of the outpatient clinic and participants and interviewers were unacquainted. Written informed consent was obtained prior to the start of the interview. The interviews lasted 1 h on average (range 44 – 85 min) and were conducted at outpatient clinic locations or at participant’s homes dependent on the participant’s preference.

### Procedure

The in-depth, semi-structured interviews were carried out by two female researchers, one psychiatrist in training (RW) and one medical student, between June and November 2016. There were no repeat interviews. Researchers were experienced in communication with anxious and depressed patients from their professional patient work and a non-judgmental interviewing style was adopted.

Interviews were guided by a topic list (Additional file 1) which was developed based on the literature and discussed in the research group which consisted primarily of science-practitioners (RW, AM, KH, TvB, NB). The topic list consisted of items concerning delay factors and barriers, facilitating factors, psychiatric history, previous experiences in mental health care and views on mental disorders and mental health care. During the course of the study, the topic list was continuously updated with new insights obtained from completed interviews.

Trustworthiness was enhanced by considering the criteria by Guba & Lincoln [27–29] and by application of prolonged engagement, persistent observation, investigator triangulation, negative case analysis, thick contextual description and reflexivity.

### Eligibility criteria

Inclusion criteria were 1) having an anxiety disorder (generalized anxiety disorder, social anxiety disorder, panic disorder, obsessive-compulsive disorder, posttraumatic stress disorder) and/or depressive disorder according to DSM-IV; 2) sufficient knowledge of Dutch or English language; 3) age ≥ 18 years. To include a diverse population and develop a broad view of participants’ current perceptions regarding treatment-seeking, no exclusion criteria were applied.

### Data analysis

The data analytical approach consisted of stepwise framework analysis to extract relevant themes. The application of framework analysis as described by Gale et al. [25] was used in this study, which is based on the framework method developed by Ritchie and Spencer [24]. This approach includes seven steps:

1. Transcription: The interviews were audiotaped and transcribed verbatim.
2. Familiarization with the interviews: One researcher (RW) read all the interviews and field notes. The other researchers read a selection of the interviews.
3. Coding: A preliminary analytical framework was developed by assigning open codes to the transcripts. MAXQDA software was used to analyse the data. Data collection and development of the preliminary analytical framework occurred in parallel. Two interviews were coded independently by two researchers (RW, medical student); subsequent interviews were line by line coded by the interviewer and verified by the other researcher.
4. Development of analytical framework: Categorization of themes and subthemes was done through discussion between the coders, and differences were discussed until consensus was reached. In monthly meetings with both interviewers and the research team (a psychiatrist, methodologist and a psychologist, all female and experienced qualitative researchers) the development of the analytical framework was discussed and adjusted, if necessary.
5. Application of the analytical framework: The analytical framework was used to analyse subsequent interviews. After 24 interviews the researchers concluded that no new themes emerged from the last interviews anymore and that data saturation was reached [30].
6. Charting data: From the analytical framework, main themes for the chart were selected by two researchers (AM, RW). These included: 1. Participant characteristics (demographics, diagnosis, treatment history, biographical context); 2. Social environment, including the role of healthcare professional and logistics; 3. Health literacy (including knowledge and disease burden); 4. Previous experiences with care; 5. Cognitions and attitudes and 6. Coping and behaviour. All members of a team of six researchers (two psychiatrists, two psychologists, a methodologist, one psychiatrist in training, 4 female, 2 male) summarized four different complete interview transcripts, resulting in 24 summaries of case descriptions. The summaries were integrated in a comprehensive chart, creating an overview of all included cases.
7. Interpreting the data: During an intensive two-day meeting, the analytical framework and chart were input for group discussions and relevant themes were discussed, compared and interpreted. Final agreement about the typology of themes resulted in a categorization of four main themes and 14 subthemes described in the Results section and Table 2.

## Results

### Participants

Twenty-four participants were interviewed and included in the analysis. Participants were between 19 and 61 years of age and 50% were female. The majority (71%) were highly educated and had a treatment history in mental health care (79%). Demographics of the participants are shown in Table 1.

**Table 1:** Characteristics of Study Sample (*N* = 24)

**Age, Mean (SD), range**	
Years	39.3 (12.5), 19-61
**Sex, N (%)**	
Female	12 (50%)
**Living alone, N (%)**	
Yes	10 (42%)
No	12 (50%)
Other	1 (4%)
Unknown	1 (4%)
**Occupation, N (%)**	
Work	13 (54%)
Student	3 (13%)
Other (e.g. sick leave; unemployed)	7 (29%)
Unknown	1 (4%)
**Educational level, higher education**^**a**^**, N (%)**	
Yes	17 (71%)
No	7 (29%)
**Chronic physical illness, self-report, N (%)**	
Yes	6 (25%)
No	15 (63%)
Unknown	3 (13%)
**Ethnic background, N (%)**	
Caucasian	16 (67%)
North African	3 (13%)
Southwest Asian	1 (4%)
Other or unknown (including adoption)	4 (17%)
**Current episode, N (%)**	
Anxiety disorder	12 (50%)
Depressive disorder	8 (33%)
Anxiety & depressive disorder	4 (17%)
**Psychiatric medication usage, N (%)**	
Yes	10 (42%)
No	10 (42%)
Unknown	4 (17%)
**Treatment history in mental health care, N (%)**	
Yes	19 (79%)
- Psychological & pharmacological treatment	10 (42%)
- Psychological treatment (including mental health nurse and family or couples therapy)	5 (21%)
- Unspecified	4 (17%)
No	5 (21%)

^a^At least 1 year of higher education (college or university)

Relevant themes emerging from the data were categorized in four main themes (individual aspects; personal social system; health care system; sociocultural background) and 14 subthemes concerning barriers and facilitators of treatment-seeking (Table 2). The most important themes are explained in the text below. Table 3 shows the pathway to care of two selected participants in detail.

**Table 2:** Main Themes and Subthemes in Treatment-Seeking Behaviour

Main themes	Subthemes	Explanation
Individual aspects	Mental health literacy	Having adequate (+) or limited (−) knowledge of mental disease and pathway to care Adequate (+) or inadequate (−) recognition of own symptoms
	Disease burden	Increasing mental distress and dysfunctioning (+), sometimes leading to habituation (−) Hindering disease characteristics; e.g. avoidance behaviour and isolation (−)
	Stigma & Attitudinal aspects	Stigma and shame (−) Negative (−) or positive (+) beliefs about oneself or about treatment
	Previous experience	Previous positive (+) or negative (−) experiences with mental health care
	Coping style	E.g. denial of symptoms (−), symptom suppression (−), solving problems alone (−)
	Physical symptoms	Somatic symptoms related to psychological disorder leading to recognition (+) or delay in psychological treatment due to somatic referral (−)
	Social problems	Housing problems (−) or occupancy with other tasks (−)
Personal social system	Recognition and encouragement from social network	Family and friends noticing (+) or not noticing (−) participants’ symptoms and encouraging treatment (+)
	Judgment from others	(Perceived) Negative social judgment about psychological complaints and treatment (−)
Health care system	Recognition and referral by professional	Correct (+) or incorrect (−) diagnosis and referral by a professional
	Therapeutic relationship with general practitioner	Having a good (+) or difficult (−) therapeutic relationship with the general practitioner
	Waiting time & Logistic barriers	Long waiting time (−) Having to find a therapist oneself (−), obstacles related to the referral process (−) and communication problems (−)
Sociocultural background	Stigma in the context of cultural background	Culture-related stigma (−)
	Media and societal influences	Information from internet or television (+) Society lacking knowledge of depression, burnout and anxiety disorder (−)

(−) indicating a barrier to treatment and (+) indicating a facilitator towards treatment

**Table 3:** Pathway to Care - Example of Two Cases

Mark^a^, male 25 years	
Mark experienced (social) phobic symptoms from the age of 8, which were interwoven with his childhood obesity. His insecurity caused by obesity in combination with the life phase he was in made it difficult to properly recognize his mental complaints; he and others attributed his withdrawal behaviour to puberty. At the age of 21 he sought treatment for the first time, suffering from problems in social contact. However, the general practitioner referred him to group therapy, which did not suit his preferences, and he never reached care but let things run their course. During this period, he lost much weight but his anxiety symptoms persisted nonetheless. Because it kept hindering him at work and studies, he started to consider seeking help again and after a year finally took this step. Facilitators were the encouragement from his partner (who was in treatment herself) and the realization that he now had enough time to commit to therapy. The general practitioner referred him to a mental health care institution, where he arrived after four months of waiting time.	
Mary-Ann^a^, female 61 years	
Mary-Ann has suffered from multiple depressions in her life and has been in treatment several times. Her first depression started in 1983 with treatment starting in 1986. For a long time, Mary-Ann thought her depressive mood was simply part of life, and only because of others’ reactions she discovered this was not normal. Her coping style was hampering as well; isolating herself and putting up a front made it difficult for others to recognize her symptoms. During her first depression, she was eventually encouraged to seek treatment by an acquaintance who managed to break through her façade. However, treatment did not succeed because the psychiatrist wanted to treat her husband and not her, even though she felt she needed treatment herself. This confirmed her negative self-image that she was a hopeless case and could not be helped. Poor experiences with mental health care affected her treatment-seeking behaviour and it took a long time before she took action again. Her belief that depression is a weakness, which was largely confirmed by her environment, also negatively influenced this process. Stigma experienced at the workplace limited possibilities to seek for help or undergo treatment during working time. For a long time she hid her symptoms and tried to keep working. She engaged in treatment in 2004, encouraged by a friend. Because of increasing disease burden, she sought treatment again in 2009 from a psychiatrist. Treatment went well, and on the advice of this psychiatrist she started group therapy, which also helped. When this therapy ended, she had to be referred elsewhere, but experienced various logistic problems causing delay. Eventually she asked for a second opinion, leading to a referral to her current therapy. Overall, Mary-Ann encountered many barriers to care but facilitating factors were the recognition by others, and at a later stage a changing attitude toward herself that she deserves care.	

^a^Fictive name

### Individual aspects

#### Mental health literacy

For many participants, some form of mental health illiteracy played an important role in treatment delay.

Participants mentioned a lack of knowledge or recognition of their psychological symptoms. Recognition was especially complicated when comorbidity masked the anxiety or depressive symptoms, such as physical comorbidity (e.g. obesity or hepatitis). Even when experiencing symptoms, participants did not always recognize these as part of a psychological disorder, nor did they acknowledge the severity of the disorder or the need for treatment. For instance, one participant did not know anything about panic attacks. When his father accidentally mentioned that panic disorder was prevalent in the family, he recognized his symptoms as panic attacks.

“I think that my father told me a story about his father, that he had panic attacks, that he had agoraphobia. I believe that was the moment that I thought: Aha, that exists!” (Male, 26 years)

Knowledge of psychological disorders made treatment-seeking easier, but only when one perceived the symptoms as a mental health problem. For example, one participant was familiar with the psychiatric classification system but did not recognize her own symptoms as obsessive-compulsive disorder (OCD). Another participant related his problems to other factors, such as stage of life (i.e. adolescence). Others said they did not recognize their symptoms as a disorder, but rather labelled the symptoms as part of their personality or personal weakness, or considered feeling down as normal:

“It is just something ( . . . ) that I am familiar with, so that is not immediately... It is just something, either way during winter I suffer from it. So it is normal to me in the first place.” (Female, 34 years)

Contrarily, some participants had good mental health literacy, which was facilitating, especially if they had experienced a similar disease episode before:

“I thought: “This is a bit like how it always starts for me, so I have to act on it early”.” (Female, 43 years)

In addition, some participants lacked knowledge about the pathway to mental health care and treatment possibilities. On the contrary, participants who were familiar with mental health care, for example because of their study, job, or previous experience, more easily found their way to treatment.

#### Disease burden

A high level of psychological distress often led to acknowledgment of a need for treatment. Some participants denied their problems or postponed treatment-seeking until the point they could not cope anymore. Furthermore, problems at school or at work caused by the disorder could be a reason to seek help.

“I thought like, I have to get rid of this ( . . . ) because it is also simply unpractical to always have to isolate yourself at work to come to yourself, to unwind.” (Male, 25 years)

Disease characteristics sometimes hindered treatment-seeking, either because the disorder itself increased avoidance behaviour, isolation and postponement of treatment-seeking, or because waxing and waning of symptoms led to delay:

“At a certain point it is so bad that you think “Wow, this is bad”, but then it gets a little better and you think: “No, I am not going to seek [treatment]”. So every time at a certain moment you think that, and then it gets better.” (Male, 26 years)

Habituation to or minimizing symptoms also delayed treatment-seeking. For instance, one participant mentioned that his panic attacks gradually deteriorated, and experiencing a new low point every week led to more habituation instead of recognizing it as a problem.

#### Stigma & attitudinal aspects

Stigma or fear of stigma was an important hindrance to treatment-seeking. Participants held beliefs that having a mental illness or having to go to a therapist is a sign of weakness. They did not want to belong to the group of persons with mental health problems, which was expressed for example by a female participant.

“I thought, that is for people who are not entirely OK, who have to go there, and I do not want to belong to them.” (Female, 35 years)

One participant who developed obsessive-compulsive disorder during pregnancy considered her psychological complaints without an understandable cause abnormal, which in combination with ridiculing statements about her symptoms from her environment made her feel ashamed.

Some mainly named feelings of shame, as they considered having mental health problems to be a sign of personal weakness or failure. Shame and fear of stigma made participants hesitant to discuss their complaints with their social network or the general practitioner. For example, one female mentioned it took a while to overcome her shame and book an appointment with her physician, because she was afraid of a negative response.

At the other end of the spectrum, some stated they did not experience any stigma, although even these participants were reluctant to share their psychological problems or their treatment experiences with others.

Apart from shame or stigma, other attitudes toward treatment influenced participants as well. One participant mentioned the fear of being medicated or hospitalized resulting in a treatment-seeking delay of 6 months*.* Participants thought therapy would not help; a few believed their complaints could not be treated because the problem was inherent to them as a person:

“Yes, because.. the feeling that it’s all on me, that I am not well and how my life is going and the problems I experience, that it is all because of how I am and that I believe that this cannot be changed.” (Female, 34 years)

For one female her negative self-image was hindering treatment-seeking, as she gathered that professionals would not know how to help her, because she was a hopeless case. This was in her eyes affirmed by a psychiatrist who decided not to treat her:

“And the psychiatrist said to me: ‘You don’t have to come here anymore, because the problem is your husband and not you”. (. . . ) And yes, I believe that I am not sane, so I am sent away, that has always been a fear for me, so that matched with my expectations. (Female, 61 years)

This experience reinforcing her negative self-perception - in addition to her self-stigma and coping style of putting up a front - caused substantial delay in further treatment-seeking:

“Researcher: And do you think it negatively influenced your motivation to seek help?

Participant: Yes, (. . .) when that first psychiatrist sent me away, I thought ‘Okay, this is it, this will have to do’. (. . . ) And then it took a long time before I started to act again [seek treatment].”

Positive attitudes toward mental health care were also reported; two participants mentioned a positive image of psychologists because of their work in the health care sector, facilitating their way to care.

#### Previous experience

For some, a negative experience in the past had a significant hampering influence. A few mentioned difficulty to discuss their problems with professionals, for example because of negative experiences with confidentiality. One male participant had a negative experience with the Child Care and Protection Board as an adolescent, making him hesitant to engage in mental health care:

”..They tried to get information from you about what was going on at home and what your father was doing and what your mother was doing. And they considered it a good idea to report back to my father about it. Well, that got back to me. And after that, there was a barrier to talk.” (Male, 58 years)

One female refused help for as long as 10 years because of her previous negative experiences. She felt inadequately treated during a clinical medication detoxification as she was discharged from the hospital and referred to her general practitioner even though she felt in crisis. During that period, she made multiple suicide attempts and experienced serious withdrawal symptoms because of sudden tapering of medication. She felt unheard by the professionals and lost hope for future treatment.

However, some participants with unfavourable or ambivalent experiences kept hope nonetheless.

Positive previous experiences encouraged future treatment-seeking. Some participants knew what help they needed because of previous episodes and were confident treatment could bring relief. For instance, one female restarted antidepressant medication by herself with approval from the general practitioner when she developed symptoms again. This participant had a smooth pathway to care, as she also recognized her symptoms at an early stage and experienced no stigma.

Some stated that previous treatment had changed their perceptions of themselves, mental illness or the mental health care system. For example, one participant said the psychiatrist positively influenced her attitude toward mental illness:

“He said to me: “This line of thought [‘if you’d be strong you wouldn’t need medication’] is really from the 60’s; your brain is simply not producing the right chemicals ( . . . ) and regarding the thought “I have to be stronger”, you don’t say that to a kidney patient either, you really have to compare it to that”. And that is one of the things that help when telling myself “this is not weak, this is something I physically need”.” (Female, 46 years)

#### Coping style

The way participants dealt with psychological problems was crucial in the process of treatment-seeking. Hampering coping styles were not showing or sharing problems, not acknowledging one’s problems, the tendency to solve problems on one’s own, substance abuse, symptom suppression and using other strategies to cope (e.g. sports). For example, one male reported that asking for help has always been very difficult for him:

“I never seek help. I don’t even dare to ask something. I always look for answers myself.” (Male, 49 years)

Other participants were used to keep on going, without reflecting on their wellbeing:

“..Just keep going without stopping and filling my agenda completely, so I do not have to think about what is going on.” (Male, 24 years)

Some had trouble acknowledging their mental problems, because it did not match their self-image, causing substantial delay. As one male participant stated:

“..and when I finally made the switch for myself ‘Okay, I cannot meet the expectations’ (. . .) and the pressure was completely gone because I finally admitted that I had problems, at that moment all barriers from the preceding six months, were all suddenly gone.” (Male, 35 years)

#### Physical symptoms

For some participants, physical problems were the first reason to seek treatment. Physical symptoms can be precursors or early signs of mental problems. It was facilitating when they were recognized as such by the patient or physician, but sometimes physical signs were interpreted as a somatic disease requiring somatic attention:

“I had lost 10 kilos of weight and my general practitioner referred me to a specialist in internal medicine, who could not find anything. Back at the general practitioner who asked the right set of questions, it became clear I was depressed.” (Male, 24 years)

One female consulted the emergency unit because of mistaking a panic attack for a heart attack.

In some cases, physical problems were easier to acknowledge than mental symptoms. One participant suffering from both mental and somatic disease, seemed to downplay his mental problems assuming it was normal that his physical disease resulted in certain (mental) problems as a side effect. This seemed to have led to delayed acknowledgment of his severe mental problems. Mainly because of the negative effect on his relationship, he saw a reason to seek treatment for his symptoms.

### Personal social system

#### Recognition and encouragement from social network

Many participants reported that recognition of symptoms by others -especially friends and relatives- contributed to their own acknowledgement and treatment-seeking.

“And then that gentleman said: “Well, if you are crying like that, I think you are not doing well”. Indeed, it was not going well, so we went to the general practitioner and notified him.” (Female, 47 years)

Acquaintances with knowledge of mental health care sometimes informed participants about treatment options. For example, one female participant mentioned that her flat mate gave her an explanation of the mental health care system, which reduced her fear and thus facilitated access to care.

In addition to problem recognition, relatives, friends or colleagues actively encouraged participants to seek help and sometimes even facilitated the path to adequate treatment. In one case, the participant’s wife made an appointment with the general practitioner for both of them, to discuss his psychological complaints. Some had a friend or relative who recommended a particular therapist or treatment. One participant reported that he would not have sought help himself, but the stimulation from others to seek treatment made him do so:

“I probably would have postponed it again; I really needed others to finally take the step.” (Male, 35 years)

Some participants were encouraged to seek help because someone they knew was successfully treated. For example, one participant was motivated by his girlfriend who was in therapy herself, and another male participant by his mother who was successfully treated in the past.

Encouragement from others to seek treatment was sometimes invigorated by social conflict. One male suffering from panic disorder had progressive avoidance behaviour, to the extent that it also hindered his partner and negatively affected their relationship, which urged him to seek help. Another participant was encouraged by a colleague because the colleague noticed she had difficulty performing at work.

Contrary to recognition, participants also reported that psychological complaints were not always visible for others, or were not understood.

#### Judgment from others

Negative attitudes from family and friends occasionally hindered participants. Participants felt their surroundings did not understand or support them, or experienced stigma from their family or colleagues. A female participant felt ridiculed by her family, which made her disguise her symptoms:

“With my family I cry a lot but they don’t care, I think. They mock you, that you cry a lot, you never laugh, you cry for everything.” (Female, 55 years)

Another participant constantly felt his father’s judgment:

“[My father] never had anything good to say about people with a similar problem as I had at a certain point.” (Male, 35 years)

Interestingly, his mother was supportive and encouraged him to seek help, thus overcoming the negative judgment from his father.

### Health care system

#### Recognition and referral by professional

For many, the general practitioner made a correct diagnosis and proper and timely referral, even in some cases when participants presented with physical symptoms. Recognition of symptoms from other professionals, e.g. a physiotherapist or midwife was facilitating as well.

However, a lack of proper recognition was also reported. One participant, who suffered from post-traumatic stress disorder (PTSD) after an accident, believed that the general practitioner should have recognized his symptoms earlier, because the general practitioner knew about the accident. His physiotherapist noticed his symptoms two years after the accident and advised him to consult his doctor. Another participant with obsessive-compulsive disorder mentioned a lack of proper referral:

“I went to the general practitioner myself and said: “I have this problem, it hurts me a lot, and I want therapy for it.” And then the general practitioner was a bit helpless, but I found out myself where I should go for help.” (Male, 45 years)

One female experienced inadequate recognition and coordination by the team from the rehabilitation centre:

“I was feeling very bad, also during (medical) rehabilitation and so they wanted me to get more psychological help, but before they figured that out it went back and forth and that took a lot of time.” (Female, 35 years)

#### Therapeutic relationship with general practitioner

Many participants considered their general practitioner accessible for discussing psychological complaints. For some participants the therapeutic alliance with their general practitioner was facilitating. The therapeutic relationship with the general practitioner is illustrative in the story of one participant of non-Western descent. Her first general practitioner -female and from the same cultural background- was approachable for her, and this physician adequately recognized her symptoms when she herself did not know what it was. Nonetheless, after being diagnosed by the general practitioner, the participant denied the problem because of stigma and hid it from friends and family. The general practitioner eventually persuaded her to seek help for the sake of her children. However, during a next episode she had much difficulty explaining her symptoms to her new general practitioner, a male Dutch doctor:

“But OK, to a man I cannot explain well what [complaints] I have. I think that he will say, “This woman is making too big a deal of things.” I try to act easy-going when I go to him. That’s the problem, I recently discovered that.”

She felt that talking about psychological complaints was whining and burdening for him. After a long delay of suboptimal treatment, a colleague eventually encouraged her to speak openly to her physician.

Several participants benefited from the long-term therapeutic relationship with their physician and the general practitioner knowing their family situation:

“Yes, and that was interesting, because he was the general practitioner who also knew my mother, and he said: ‘I have been waiting and I was wondering when you, the children, would come here, because you probably have a difficult time’. And I said: ‘I am so incredibly tired, should we do a blood test?’. And then he said; ‘Well, I won’t mind doing that, but you are basically depressed’ (Female, 46 years)

#### Waiting time & logistical barriers

Participants reported additional delay factors after having taken the first step but before the start of treatment. Primarily, many participants experienced a substantial waiting period, leading to much dissatisfaction.

“There are moments that you don’t want to do much about it, your panic attacks and your symptoms and solving your problems, and there are certain moments when you really want to do something about it and then you start to call and take action. These are exactly the moments that are very important and to hear then that there is a 10-week waiting period, that is annoying, yes.” (Male, 26 years)

Another participant stated that a long waiting period might even make her drop out. Some experienced delay or never reached care during a previous episode because they had to find a therapist themselves after being referred, or the proposed treatment was not in line with the participants’ preference. For instance, one participant had not effectuated the referral because he did not agree with the type of treatment. It took him four years after that to consult his general practitioner for help again.

Quite a few participants reported hindering logistic factors; problems concerning the referral process; suboptimal communication and care in case of comorbid substance abuse disorder and difficulty contacting mental health care institutions. Financial reasons were of minor importance, though mentioned by one young male participant who discontinued previous treatment because of the costs.

### Sociocultural background

#### Stigma in the context of cultural background

Cultural aspects appeared to be hindering in some cases:

“In my culture [North African], it is seen as somewhat normal to be depressed. They say: You are dramatizing, you just want to escape your duties when you complain about depression. Admit you just do not want to cook or clean the house.” (Female, 55 years)

On the other hand, another participant with a North African background noticed positive societal changes concerning stigma, stating it is seen as normal to visit a psychologist for mental problems, in the Netherlands as well as in her country nowadays. However, she still seemed to experience a taboo to openly talk about mental problems with others.

“No I am not afraid; still I am not going to tell [anyone]. I am not afraid, everybody is seeing a therapist, everybody here. In [country in North Africa], that is normal. For the first-generation, [if you see] a psychologist means you are crazy. But the youth is also going to a psychologist in [country in North Africa].” (Female, 52 years)

#### Media and societal influences

One male mentioned that not friends or relatives, but TV-series, books or internet informed him about psychological problems:

“There is a reason that I started searching the internet because there was nothing on media like television, advertising campaigns. ( . . . ) A very clear TV-series, is The Sopranos, they handle the topic very well. That the problem exists and that you can experience it, [I realized that] because of this series.” (Male, 26 years)

A few participants mentioned that society lacks knowledge of depression, burnout and anxiety disorder and some believed there should be more attention for these disorders in the media.

## Discussion

This study reports on barriers and facilitators of treatment-seeking in individuals with an anxiety or depressive disorder in a Western-European outpatient mental health care setting. With the use of a qualitative study design, rich in-depth information was gathered, leading to deepening insight on factors influencing treatment-seeking and actually receiving treatment.

Our findings indicate that treatment-seeking reflects a complex interplay of multiple factors. We found mental health illiteracy, negative attitudes toward professional help -including stigma- and logistic problems to be prominent barriers to care. Social networks and the general practitioner were often helpful to access care.

Although qualitative studies on treatment-seeking behaviour have been carried out previously [13, 17, 31–33], limited information is available for the European population other than the UK. This study describes the barriers and facilitators among Western-European individuals, thus adding to the existing literature. When interpreting our findings and comparing them with former research some results stand out.

Remarkably, substantial stagnation seems to occur early in the treatment-seeking process often because of mental health illiteracy and stigma. Mental health literacy is generally defined as “knowledge and beliefs about mental disorders which aid their recognition, management or prevention [34]. Even in this study from a developed country in a sample with predominantly highly educated individuals, mental health illiteracy was reported by many participants as a crucial factor in patient delay. Mental health illiteracy has been previously found to be an important barrier, both in patients with depressive disorder, OCD and anxiety disorders [14–17, 20]. Apart from limited knowledge of symptoms, not knowing where to find help was another barrier reported in our study, in accordance with other Dutch and Australian samples [14, 19, 21]. These findings indicate that improving mental health literacy is still a major concern and may be one of the factors that can be influenced to improve treatment-seeking. Previous studies have shown promising results of interventions aimed at improving mental health literacy [35–37]. Furthermore, adequate mental health literacy and recognition of symptoms seems to positively influence treatment-seeking from formal sources [38] and mental health care use [39]. However contradicting results have been found as well. Previous literature, including a systematic review on web-based interventions [35, 40] could not confirm a positive effect of mental health literacy on actual treatment-seeking behavior. This suggests that multiple factors play a role in treatment-seeking, which is in line with the results from our study.

Indeed, we found that attitudinal factors greatly influence treatment-seeking behaviour as well, which is in line with previous research [12–14, 17–22]. Many participants described shame or stigma, including both personal stigma and stigma experienced from their social network or surroundings. It is noteworthy that there appears to be some form of concealed stigma, as some participants stated not to experience any stigma while simultaneously being hesitant to disclose their symptoms or their treatment-seeking to others. Only a minority of participants reported no stigma and spoke openly about their mental problems, for example because other people from their (urban) subculture were receiving mental health treatment. A meta-analysis [41] on the association between stigma and treatment-seeking found that negative attitudes either towards treatment-seeking or towards subjects suffering from mental illness were significantly and negatively related to active treatment-seeking. Also, self-stigma (i.e., negative attitudes towards one’s own mental symptoms) was related to less treatment-seeking -albeit nonsignificantly-, but no relation with public stigma (defined as “the individual’s perception of public stigma”) was found. The results from Schnyder [41] are predominantly in line with our findings, although we found public stigma to influence treatment-seeking as well. Concerning self-stigma, previous studies [13, 32] postulated that individuals avoid seeking help because acknowledging one’s depressive symptoms leads to a potential identity threat; negative consequences for their integral identity and their main roles. Indeed, in our study participants experiencing stigma commented negatively on their self-perception, e.g., considering oneself weak because of depressive symptoms or not wanting to be ‘one of those people’ who need therapy. Although it was not the case for all participants, some had much difficulty acknowledging the symptoms and their impact, only accepting it when it could no longer be ignored. It has been suggested [35] that stigma is one of the main factors hampering treatment-seeking in individuals with improved mental health literacy. Thus, it is of great importance to reduce stigma. Interventions aimed at stigma reduction have shown promising results, although their long-term effects are less clear [42].

The Dutch insurance system provides almost full coverage for mental health care. Correspondingly, in our study financial barriers played a minor role, contrasting studies from other countries [12, 16, 20, 21, 43–45]. Similar to several other European countries, the Netherlands has a high-access healthcare system, with a strong and accessible primary health care system [2]. Furthermore, a mental health nurse is available in most primary care centres. However, access barriers were present even in this setting. Problems related to the referral and in particular waiting time for mental health care professionals or institutions after being referred were frequently mentioned and counted for significant additional delay. This is unfortunate because individuals often had to overcome substantial hindrances (e.g., mental health illiteracy or stigma) in order to seek assistance, and institutional barriers thereafter can be detrimental to their fragile motivation to seek help. A variety of logistical barriers, including waiting time, transportation problems and lack of accessibility have been reported before and seem to differ per country [12, 14, 16, 20, 45]. This stresses the extent and relevance of this problem, and the importance for governments and institutions to address the specific organisational aspects that account for delay in their particular setting.

In our population only a few participants had a remarkably easy way to care; in these cases one crucial factor was correct and early recognition of symptoms because of health literacy and/or significant disease burden. Furthermore, positive previous experiences with mental health care were important in swift treatment access. These insights into successful pathways to care are an addition to the existing literature and of relevance since it yields information on the crucial themes involved in treatment-seeking.

Given the aforementioned barriers and the complexity of the treatment-seeking process, focus on potential facilitators is crucial. Barriers early in the treatment-seeking process could be overcome by facilitators such as recognition from others. In our sample, recognition of symptoms by friends and relatives positively influenced participants’ own realization and acknowledgement of mental disease, thus enabling treatment-seeking. Moreover, encouragement, specifically from family or friends, was pivotal in some cases to seek help. Conversely, negative judgment from others could reinforce stigma and was hampering in acknowledging the need for help. Hence, it seems that having a knowledgeable, well-informed and supportive social network is crucial particularly for people experiencing mental health illiteracy or stigma. People with a non-supportive social support system might therefore be a vulnerable subgroup that could experience more detriment. The positive impact of social surrounding has been previously found in adults [13, 17] and young adults specifically [46]. In addition to positive encouragement, personal conflicts were reported as negative enablers; e.g., conflicts at work or with one’s partner urged participants to seek treatment. This is in line with results from a quantitative Japanese study [47], which found interpersonal problems to be positively associated with treatment-seeking. To improve support and recognition of mental health illnesses from the community, interventions have been developed, e.g., Mental Health First Aid [48]. This intervention teaches lay individuals how to recognize mental symptoms in others and how to lead them to proper treatment [49]. The intervention leads to increased knowledge and recognition and reduced stigma in individuals who completed the intervention, and a small increase of provided help to individuals with mental health problems [49].

Furthermore, we found the general practitioner’s role to be important. In many cases, the general practitioner positively affected the pathway to care because of proper recognition of symptoms, encouragement to seek treatment and timely referral. This is a new finding, as contrary to our results, a systematic review [13] mainly reported general practitioner-related barriers; i.e. the belief that they have limited expertise regarding depression treatment, little intimacy or empathy from the general practitioner and problems discussing one’s symptoms with the physician. This supposed contradiction might be explained by the fact that in our sample all participants eventually did access mental health care and the fact that in the Netherlands the general practitioner plays a key role in health care with approximately 75% of patients being content about their general practitioner [50]. However, also in our sample a few participants were dissatisfied with the role of their physician. Undoubtedly, the general practitioner is an important gatekeeper in mental health care who can either facilitate or hinder treatment-seeking.

To structure the extensive amount of factors relevant to treatment-seeking found in the literature, several theoretical frameworks have been developed in the past. Among the most widely used theoretical frameworks are the Behavioural Model of Health Services Use [51], the Health Belief Model [52], the Theory of Planned Behaviour [53], and the Network Episode Model (NEM) by Pescosolido [54]. When considering the findings of our study, the Network Episode Model seems to be the best applicable theoretical framework.

The NEM focuses on the entire illness pathway, or so-called illness career. It identifies several subsystems that play a role in health behaviour and treatment-seeking: the community systems, the institutional systems, the support (personal network) systems, the individual (self and body) and, according to the latest version of the model, the molecular system. Although the NEM validates the importance of explanatory factors from previous theories, it places most emphasis on the role that social networks have in an individual’s health behaviour [54].

The four main themes from the present study were largely in accordance with the NEM [54], although we labelled them slightly different. Our fourth theme ‘sociocultural background’ (see Table 2) could be considered similar to the NEM’s community system, although ‘sociocultural background’ in our study primarily reflects influences from the community (e.g., experienced stigma or the media), whereas the NEM seems to take a more geographical approach towards community (with a subdivision in global, national, regional, political divisions, community and neighbourhood). The factor ‘health care system’ in this study (see Table 2) overlaps with the institutional systems explained by the NEM, which comprises the treatment system, professional organizations and civic and religious organizations. Our main theme ‘personal social system’ (see Table 2) corresponds to the NEM’s personal support system, which includes family, friends, work and voluntary organizations. Lastly, the ‘individual aspects’ found in our study (see Table 2) largely correlate with the individual system of the NEM.

However, discrepancies with the model do exist. First, we did not identify a factor that corresponds to the molecular system as described by the NEM. Second, our research only investigated treatment-seeking behaviour and did not aim to describe the entire illness career. Third, some subthemes might be categorised differently according to the NEM. For instance, the logistic barriers of the health care system in our study are not primarily social in nature, and thus it remains unsure if they should be categorised in the institutional system of the NEM. Furthermore, the NEM places most emphasis on the influence of social networks. Our study however, found an important role for individual factors as well (including determinators such as mental health illiteracy and health beliefs) that do not seem to have a prominent role in the NEM. Lastly, the NEM describes a highly elaborate model with many details, themes and correlations that could not all be confirmed in our study. Nonetheless, in the light of improving comparisons across studies, the NEM may provide a valuable model to categorise findings of (future) studies on help-seeking.

### Strengths and limitations

One strength of this study is its qualitative design, yielding an extensive amount of information on treatment-seeking behaviour. Furthermore, our study focused both on barriers and facilitating factors, which are not necessarily opposites and important to investigate separately [14]. In contrast with previous studies, which mostly focused on patients with depression, we included participants with anxiety disorders as well.

Much effort was put in recruiting a diverse study population, resulting in a well-balanced study sample with regard to age, gender, and psychiatric history. Although recruiting participants from non-Western descent is known to be difficult, we included 25% participants from non-Western descent. Nonetheless, as most participants in our sample were highly educated, views of people with limited education might be relatively underrepresented. Furthermore, since participants were invited by their therapist, the relationship with the therapist may have influenced their willingness to participate and we lack information about patients who refused to participate*.* Lastly, we only recruited participants who managed to access mental health care. It is possible that other barriers and facilitators play a role in individuals with anxiety and depression who did not reach mental health care at all, or for those who sought help from other sources than mental health care institutions. Consequently, we may have missed relevant barriers and facilitators to treatment-seeking in the population. Future studies should focus on these specific subgroups.

### Implications

Our findings emphasize that it might be helpful to educate the general population by means of national public health care campaigns about mental health problems and stigma and to propagate disclosure of symptoms. Research has shown national campaigns to be effective in improving depression awareness and health literacy [36, 37]. Based on our finding that the social environment plays a substantial facilitating role such campaigns may specifically empower the social environment as well. Furthermore, in our sample previous experience influenced treatment-seeking significantly. Former research has shown that therapy adherence, satisfaction with treatment and outcome of treatment are better if therapy is adjusted to patients’ preferences [55]. These findings emphasize the need to involve patients in therapy decision-making in order to improve treatment satisfaction and outcome and hence positively influence future treatment-seeking.

In addition to hindrances early in the treatment-seeking process, subsequent barriers could be addressed by health care providers. Mental health care institutions should thoughtfully try to diminish practical barriers –in particular waiting time- encountered directly prior to treatment. Health care professionals should be sensitive to psychological problems, also in patients with physical symptoms, and be aware of stigma and mental health illiteracy in their patients. Finally, both the general practitioner and mental health care organisations should feel responsible to clarify what to expect from mental health treatment and exercise shared decision-making.

## Conclusion

Extending previous research from different countries, this study provides information on treatment-seeking behaviour in a Western-European setting. Financial barriers for treatment-seeking were of minor importance. However, health illiteracy and stigma were frequently encountered and disrupted early stages of treatment-seeking behaviour. For many participants, the social network and healthcare providers accelerated treatment-seeking. Barriers encountered in later stages consisted of practical aspects, including waiting lists and logistic problems.

Education of the general population is required to facilitate timely treatment and to ameliorate the prognosis of anxiety and depressive disorders. Concurrently, adequate training in mental health of general practitioners and the management of waiting lists should be prioritised to improve access to care.

## Supplementary Information

**Additional file 1.** Topic list

## Data Availability

The datasets generated and/or analysed during the current study are not publicly available due to privacy restrictions but are available from the corresponding author on reasonable request. All interviews were conducted in Dutch and quotations were translated to English.

## Abbreviations

COREQ: Consolidated criteria for reporting qualitative research
DSM: Diagnostic and statistical manual of mental disorders
OCD: Obsessive-compulsive disorder
PTSD: Post-traumatic stress disorder
US: United States
UK: United Kingdom
VU: Vrije Universiteit
WHO: World Health Organization
