# Supplementary material for: Barriers and facilitators for treatment-seeking in adults with a depressive or anxiety disorder in a Western-European health care setting: a qualitative study
Source: BMC Psychiatry. 2022 Mar 5;22:165. doi: 10.1186/s12888-022-03806-5 (PMC8898419; doi:10.1186/s12888-022-03806-5)
Supplement: Supplementary file 1 — Additional file 1. Topic list [file 12888_2022_3806_MOESM1_ESM.docx]

**Additional file 1 Topic list**

At the start of the interview personal information was assessed including relational status, daily occupation/work status, education level, (chronic) physical illness, ethnicity, current and previous diagnoses, current treatment and medication usage.

1. How was your pathway to mental health care?

[e.g., how long did it take to seek and enter treatment, treatment-seeking on own initiative or someone stimulated treatment-seeking, contact with the general practitioner]

What were barriers to seek help?

[e.g., not noticing or recognizing symptoms, seeing symptoms as “normal”, trying other ways to diminish symptoms]

What prompted you to seek help?

[e.g., severity of symptoms, deterioration in functioning]

2. In case you experienced previous episodes of mental distress for which you received treatment: how did you experience this previous episode and the treatment you received?

3. Which role did friends, family or partner play in the process of treatment-seeking?

4. Which role did health care professionals play in the process of treatment-seeking (general practitioner, hospital physicians, psychologists, professionals in mental health care institutions and other professionals such as physiotherapists)?

Did you know where to find help?

5. What do you think of mental health care?

How do you feel about psychotropic medication and psychotherapy?

6. Do you think your symptoms play a role in the treatment-seeking process?

[e.g., not having the motivation to seek help in case of depression, or being afraid to discuss your symptoms because of social anxiety]

7. Which role do stigma and shame play in seeking treatment?

[e.g., views of patient and social network on individuals with a mental disorder, experiences with or ideas about sharing about mental problems with social network, ideas about the personal consequences of having a psychiatric diagnosis]

8. How do you normally deal with problems and misfortune?

9. Which role did the media play in your decision to (not) seek treatment?

10. Are there factors that could have helped you to seek treatment at an earlier stage?

11. Which other barriers hampered treatment-seeking?

[e.g., financial aspects, occupancy with other tasks]
